# Burden of subclinical heart and lung disease detected on thoracic CT scans of HIV patients on HAART

**DOI:** 10.7448/IAS.17.4.19716

**Published:** 2014-11-02

**Authors:** Stefano Zona, Antonella Santoro, Giulia Besutti, Guido Ligabue, Cristina Mussini, Paolo Raggi, Jonathon Leipsic, Don D. Sin, Giovanni Guaraldi

**Affiliations:** 1 Infectious Disease Clinic, AOU Policlinico di Modena, Modena, Italy; 2 Radiology, AOU Policlinico di Modena, Modena, Italy; 3 Mazankowski Alberta Heart Institute, Edmonton, Canada; 4 Radiology, University of British Columbia, Vancouver, Canada; 5 Medicine, University of British Columbia, UBC James Hogg Research Center, Vancouver, Canada

## Abstract

**Introduction:**

The aim was to determine the prevalence of lung and heart abnormalities on thoracic CT scans in HIV-infected patients who were treated with antiretroviral therapy (ART).

**Material and Methods:**

Thoracic CT scans of 903 patients infected with HIV (mean age 48±7 yrs, 29% females) were reviewed by three radiologists by consensus. Patients were phenotyped according to smoking status, pack years and years since cessation for ex-smokers. Individuals known to have active lung or heart disease at the time of CT scanning were excluded. Multimorbidity lung and heart disease (MLHD) was defined by the presence of >2 lung or heart abnormalities on the CT scan.

**Results:**

Prevalence of lung abnormalities were: 326 patients (36.1%) with emphysema, 271 (30.0%) with bronchiolitis, 44 (4.9%) with non-calcified lung nodules, 568 (63%) with significant bronchial wall thickening, 150 (16.7%) with bronchiectasis, 9 (1%) with interstitial lung disease. Overall, 445 patients (49.3%) had >2 lung abnormalities. Imaging findings suggestive of prior myocardial infarction (MI) were found in 1.4% (13 patients); 26.6% (240 patients) had CAC scores of 1 to 100, and 9.8% (89 patients) had CAC>100. 13.6% (123 patients) of the patients had CAC>100 and/or previous MI. MLHD was present in 484 patients (53.6%) and among 78 patients (16%) who never smoked. Table 1 describes CT findings according to pack year and stop smoking groups vs never smokers.

MLHD increased proportional to cumulative smoking history (p for trend <0.001) and decreased in proportion to the number of years since smoking cessation (p for trend=0.017). Independent predictors for MLHD were: age (OR=1.07, CI 1.05–1.10), sex (OR=1.59, CI 1.15–2.19), current smoking (OR=1.76, CI 1.08–2.89), and pack-years history of smoking (OR=1.03, CI 1.02–1.05). In patients who never smoked, nadir CD4<200 was significantly associated with MLHD after adjustment for age and sex (OR=1.98, CI 1.98–3.63).

**Conclusions:**

MLHD is common in HIV-infected individuals even in non-smokers. Reduced CD4 count (hence severity of HIV infection) may be an important risk factor for chronic lung and heart disease. Thoracic CT scans may provide an excellent screening tool to detect MLHD in HIV-infected patients.

**Table 1:** Prevalence of lung and heart CT abnormalities according to cumulative smoke exposure and according to time since interruption

	Never smoker		Pack year group		p-value (p per trend)	Never smoker		Stop smoking group		p-value (p per trend)
		0–10	11–20	>20			<10 years	10–20 years	>20 years	
Emphysema	41 (19%)	37 (23%)	62 (34%)	186 (54%)	<0.001 (<0.001)	41 (19%)	35 (33%)	25 (27%)	24 (39%)	0.003 (0.01)
Bronchiolitis	27 (13%)	31 (19%)	57 (31%)	156 (45%)	<0.001 (<0.001)	27 (13%)	18 (17%)	15 (16%)	14 (23%)	0.24 (0.29)
Lung nodules	15 (7%)	8 (4.94)	5 (2.73)	16 (4.65)	0.26 (0.19)	15 (7%)	3 (2.9%)	5 (5.4%)	2 (3.28%)	0.39 (0.14)
Bronchial wall thickening	104 (49%)	83 (52%)	119 (65%)	262 (76%)	<0.001 (<0.001)	104 (49%)	65 (62%)	49 (53%)	40 (65%)	0.04 (0.05)
Bronchiectasis	33 (16%)	21 (13%)	35 (19%)	61 (18%)	0.42 (0.29)	33 (16%)	18 (17%)	13 (14%)	17 (28%)	0.11 (0.97)
Interstitial lung disease	1 (0.5%)	0 (0%)	1 (0.6%)	7 (2%)	0.09 (0.03)	1 (0.5%)	0 (0%)	1 (1%)	0 (0%)	0.6
Multimorbidity lung	65 (30%)	55 (34%)	92 (50%)	233 (68%)	<0.001 (<0.001)	65 (30%)	47 (45%)	34 (37%0)	32 (52%)	0.005(0.02)
Myocardial infarction	9 (4%)	4 (2.5%)	9 (5%)	25 (7%)	0.11 (0.048)	9 (4%)	13 (12%)	5 (5%)	6 (9%)	0.039 (0.02)
CAC >100	16 (7.5%)	8 (5%)	17 (9%)	49 (14%)	0.004 (0.002)	16 (7.5%)	9 (8%)	13 (14%)	8 (13%)	0.25 (0.42)
Multimorbidity heart	24 (11%)	10 (6%)	23 (13%)	66 (19%)	<0.001 (<0.001)	24 (11.%)	18 (17%)	15 (16%)	12 (19%)	0.26 (0.1)
Multimorbidity lung and heart disease (MLHD)	78 (36%)	59 (36%)	98 (53%)	249 (72%)	<0.001 (<0.001)	78 (36%)	55 (52%)	38 (41%)	33 (54%)	0.013 (0.017)

